# Cross-cultural validation and psychometric evaluation of the revised emancipated decision-making scale in Portuguese pregnant women

**DOI:** 10.1016/j.pecinn.2026.100471

**Published:** 2026-03-15

**Authors:** Marlene Isabel Lopes, Teresa Margarida Almeida Neves, Ruth A. Wittmann-Price, Margarida Vieira, Alexandrina Cardoso

**Affiliations:** a Universidade Católica Portuguesa, Centre for Interdisciplinary Research in Health: Faculty of Health Sciences and Nursing, Porto, Portugal; b Health Sciences Research Unit: Nursing (UICISA: E), Nursing School of Coimbra (ESEnfC), Coimbra, Portugal; c W. Cary Edwards School of Nursing and Health Professions, United States; d Nursing School of Porto (ESEP), CINTESIS@RISE: Center for Health Technology and Services Research, Porto, Portugal

**Keywords:** Decision making, shared, Women's health, Prenatal care, Empowerment, Psychometric

## Abstract

**Objective:**

Active participation in healthcare decisions during childbirth is strongly associated with greater satisfaction and improved health outcomes. Shared decision-making is particularly critical in women's health, where choices have often been shaped by patriarchal norms and a historically paternalistic healthcare system. This study aimed to evaluate the psychometric properties of a culturally adapted Portuguese version of the Revised Emancipated Decision-Making Scale (EDMr) for use among pregnant women.

**Methods:**

A two-phase methodological approach was employed, involving the cultural adaptation of the instrument and the assessment of its psychometric properties in a non-probabilistic sample of 241 pregnant women in Portugal. Exploratory Factor Analysis and Confirmatory Factor Analysis were performed on separate subsamples to enhance methodological rigor.

**Results:**

The original three-factor model evolved into a five-factor structure: Personal Knowledge, Flexible Environment (subdivided into Feeling Supported and Feeling Respected), and Social Norms (subdivided into Sense of Pressure and Negative Reactions). The final model demonstrated solid psychometric properties, with acceptable to high internal consistency across subscales, with Cronbach's alpha values ranging from 0.68 (Sense of Pressure) to 0.86 (Personal Knowledge).

**Conclusion:**

The findings support its theoretical soundness and cultural relevance, confirming its utility in assessing women's autonomy in healthcare decision-making during pregnancy and childbirth. Further research is recommended to explore its applicability in broader populations and across diverse clinical contexts.

**Innovation:**

This represents the first validation of the EDMr outside the United States.

## Highlights

• First EDMr validation outside United States confirms strong cross-cultural relevance.
• Revised five-factor model shows solid psychometrics in Portuguese sample.
• EDMr-PT assesses emancipated decision-making among Portuguese women.
• Supports research, clinical, and cross-cultural use in women's health decisions.

## 1 Introduction

Decision-making in healthcare has emerged as a central focus in nursing, particularly with the growing emphasis on shared decision-making and person-centered care. Introduced in 1982, shared decision-making is grounded in ethical principles of respect for autonomy and self-determination [1]. This approach holds particular relevance in women's health, a field where healthcare decisions have historically been influenced by patriarchal norms and a paternalistic system that frequently marginalized women's voices [[2], [3], [4], [5]]. Perinatal care offers a vivid example of how societal structures influence healthcare practices, often reflecting traditional gender roles [6]. During pregnancy, childbirth, and the postpartum period, women frequently face complex, preference-sensitive decisions, situations where multiple medically appropriate options exist or where clinical uncertainty prevails. In these scenarios, decision quality depends not only on clinical evidence but also on women's preferences and understanding of associated risks and benefits [7]. Research consistently shows that involving women in childbirth decisions improves maternal and neonatal outcomes and enhances satisfaction with the birth experience [[8], [9], [10]].

Despite this evidence, healthcare systems often maintain entrenched models that prioritize paternalism over person-centered care, limiting women's autonomy. Although self-determination is widely recognized as a fundamental right, conservative societal norms frequently obscure the impact of sociocultural factors on women's health [[11], [12], [13], [14]]. Globally, significant disparities in women's autonomy persist, carrying serious implications for both their health and the well-being of their families. According to the United Nations Population Fund [15], the proportion of women empowered to make healthcare decisions ranges from below 40% in Central and Western Africa to nearly 80% in parts of Europe, Southeast Asia, Latin America, and the Caribbean.

Self-efficacy is a key enabler of women's autonomy, empowering them to act as agents of change in their healthcare decisions [16]. Women with prior childbirth experience, strong self-esteem, and robust social support networks often demonstrate greater decision-making capacity. However, structural barriers, such as prolonged waiting times, staff shortages, and restrictive sociocultural norms, often impede women's ability to exercise autonomy in perinatal care decisions [17]. While many women express a desire to be actively involved in decisions related to pregnancy and childbirth, this aspiration is often unmet. In some contexts, healthcare professionals adopt paternalistic practices, offering limited information or options, which undermines informed consent [[18], [19], [20]]. In Ireland, for instance, contextual and relational challenges, such as professional unavailability and pressure to accept interventions, have been shown to constrain women's participation in labor-related decision-making [21]. Healthcare professionals themselves recognize the impact of systemic pressures that prioritize efficiency over individualized care, influencing clinical practices and communication styles, and thereby increasing women's vulnerability in maternity care settings [22].

Effective implementation of shared decision-making in perinatal care requires not only adequate time but also a supportive environment in which women feel safe to express their values, preferences, and choices regarding childbirth. In busy, understaffed hospital settings, several specific barriers, such as lack of continuity of care, inconsistent hospital cultures, absence of clear policies or protocols, and a lack of consensus on the definition and practical application of shared decision-making, present significant challenges. Continuity of care, in particular, is critical for fostering trust between women and healthcare providers. However, in many perinatal care settings, continuity remains uncommon: most women receive antenatal care from community-based providers, while intrapartum care is delivered by an unfamiliar team at the time of birth [23].

The concept of emancipated decision-making, developed within nursing, addresses these challenges by promoting autonomy and freedom in healthcare decisions, particularly in contexts where women experience emotional or systemic oppression. Wittmann-Price [5] defines emancipated decision-making as a process through which individuals come to perceive their choices as truly optimal for their own needs. The Theory of Emancipated Decision-Making in Women's Healthcare builds upon empowerment and shared decision-making frameworks, integrating principles from critical social theory, feminist perspectives, and Paulo Freire's emancipatory education. It emphasizes women's active engagement in their own care, grounded in access to knowledge, awareness of social norms, and the creation of supportive and flexible healthcare environments that foster authentic and autonomous choices. These principles reflect key dimensions of empowerment and shared decision-making, which value partnership, critical reflection, and informed participation in the care process. Together, these elements contribute to greater satisfaction and a stronger sense of agency in decision-making [24].

The EDM Theory has been empirically supported through four studies conducted in the United States, exploring decision-making across diverse clinical contexts, including infant feeding [25], pain management during labor [6], delivery mode [26], and medication use for anxiety or depression during pregnancy [27]. These studies consistently demonstrated positive correlations between emancipated decision-making and satisfaction with decisions, with personal knowledge and a flexible environment emerging as the strongest predictors of satisfaction. Across these studies, the original 35-item EDM Scale was progressively refined through empirical testing in different clinical contexts, resulting in the 20-item Revised Emancipated Decision-Making Scale (EDMr), composed of three subscales: Personal Knowledge, Social Norms, and Flexible Environment. Internal consistency coefficients ranged from α = 0.84 to 0.91 across the developmental studies, reflecting progressive improvement as the scale was streamlined. In the final U.S. version of the EDMr, a reliability coefficient of α = 0.89 was reported, demonstrating strong internal consistency and theoretical coherence [28].

Nurses play a vital role in facilitating this process by using therapeutic communication and recognizing women's experiential knowledge as valid and trustworthy. To implement shared decision-making effectively, healthcare professionals must identify patient preferences, address socially embedded norms, and create environments that enable autonomous decision-making [26]. Identifying women at risk of compromised decision-making capacity is also essential for designing targeted interventions to enhance both decision-making and satisfaction with care.

This study aims to evaluate the psychometric properties of the European Portuguese version of the EDMr for use among pregnant women. The scale was culturally adapted and validated for the Portuguese context. Its validation is expected to provide deeper insights into women's decision-making processes and the factors that influence them. These insights may contribute to advancing nursing and midwifery knowledge, supporting evidence-based practice, and informing the development of decision-making support strategies that promote person-centered care, improve health outcomes, and enhance women's satisfaction with maternity services.

## 2 Methods

### 2.1 Design

This methodological study was conducted in two phases: 1) Cross-cultural adaptation of the original version; 2) Evaluation of the psychometric properties of the Portuguese version.

#### 2.1.1 Cross-cultural adaptation

The cross-cultural adaptation of the scale followed the five-step process outlined by Beaton et al. [30]. Initially, a comprehensive literature review was conducted to explore the concept under study, supplemented by theoretical discussions with a panel of experts. The discussions focused on assessing the relevance of each item for evaluating the concept of emancipated decision-making and its applicability to the context of pregnancy and childbirth healthcare in Portugal. Authorization to translate, adapt, and validate the scale for use in European Portuguese was obtained from the original author.

Step 1 - Translation: Following the author's permission, two independent native Portuguese translators conducted separate translations of the original English instrument into Portuguese, focusing on achieving semantic equivalence.

Step 2 - Synthesis: The research team collaborated with the translators to review both translations and reach a consensus on each item. Discrepancies were minor, primarily related to grammatical structures or synonyms with equivalent meanings in Portuguese.

Step 3 – Back translation: Two independent native English-speaking translators, unfamiliar with the original scale, performed back-translations of the Portuguese version into English. This step confirmed linguistic equivalence between the adapted version and the original scale.

Step 4 - Expert Panel: The expert panel comprised two doctoral-level nursing researchers with expertise in methodological research and one midwife with a master's degree and over 20 years of clinical experience in midwifery care. The panel conducted a detailed comparison of the translations, back-translations, and the original scale to evaluate the cultural and contextual relevance of each item for Portuguese pregnant women. Key aspects assessed included conceptual and semantic equivalence, idiomatic expressions, experiential relevance, and overall readability.

Step 5 - Pretest: Version 1 was pretested with 18 pregnant women enrolled in a childbirth preparation program at two selected healthcare units. These participants met the inclusion criteria. Under the guidance of the principal investigator, participants completed the instrument and provided feedback on its comprehensibility. Follow-up interviews were conducted to assess the clarity of the expressions, semantic accuracy, and experiential relevance of the scale items, serving as an evaluation of face validity.

#### 2.1.2 Psychometric properties

##### 2.1.2.1 Participants

A convenience sample of 241 pregnant women enrolled in Childbirth Preparation Programs (CPPs) at nine Community Care Units (CCUs) in central Portugal was recruited for this study over a one-year period, from September 2023 to September 2024. Given that the EDMr consists of 20 items, and following the recommended participant-to-item ratio of 10:1, a minimum sample size of 200 was deemed necessary for validation [31]. Ethical approval was obtained from the “XX” (CE “XX”/no. XX/202×). Inclusion criteria were pregnant women aged 18 years or older with a low-risk pregnancy. Exclusion criteria included an inability to understand or speak Portuguese and a planned cesarean birth. The principal investigator presented the study objectives to nurse-midwives at participating CCUs and secured their agreement to assist with participant recruitment. Eligible participants were invited during CPP sessions by the attending nurse-midwife, who explained the study objectives and emphasized that participation was voluntary and would not affect the quality of their care. Women who consented to participate received detailed instructions and were given the option to complete the questionnaire either on paper, sealing their responses in an opaque envelope, or online via a QR code provided in the study materials. Informed consent was obtained either in paper form or electronically, corresponding to the chosen method of questionnaire completion.

##### 2.1.2.2 Instruments

The questionnaire comprised two sections. The first section gathered sociodemographic and obstetric data, including age, educational level, employment status, cohabitation with the baby's father, gestational age and previous births. The second section consisted of the *Revised Emancipated Decision-Making Scale* (EDM-r), a 20-item instrument using a 5-point Likert scale ranging from “strongly disagree” (1) to “strongly agree” (5). The scale evaluates women's capacity to make emancipated decisions regarding their health, with total scores ranging from 20 to 100. Items 2, 4, 8, 9, 15, and 16 are reverse-scored, reflecting negative statements. Women scoring above 4.0 on the total scale are considered to make emancipated decisions and report satisfaction with their choices. The original scale has demonstrated strong reliability (Cronbach's alpha = 0.89) and includes three dimensions: Personal Knowledge (6 items; Cronbach's α = 0.78), Flexible Environment (7 items; Cronbach's α = 0.74), and Social Norms (7 items; Cronbach's α = 0.77).

##### 2.1.2.3 Data analysis

The total sample was randomly divided into two independent subsamples using the random case selection procedure in SPSS to conduct the Exploratory Factor Analysis (EFA) and Confirmatory Factor Analysis (CFA). Random allocation was applied without stratification. Therefore, equivalence between subsamples regarding participant characteristics was not controlled. Given that this was the first cross-cultural validation of the EDMr, an exploratory-confirmatory sequence (EFA followed by CFA) was adopted to examine whether the original factor structure would be replicated in the Portuguese context and to explore potential cultural variations in item behavior.

The Portuguese version of the EDMr underwent EFA to investigate the underlying structure of the items. Factor extraction was performed using the Principal Axis Factoring method with Promax rotation, as the latent dimensions were theoretically expected to correlate. Given the five-point Likert response format and the absence of substantial deviations from normality, the analysis was based on the Pearson correlation matrix. Factor retention was guided by multiple criteria, including eigenvalues greater than one, inspection of the scree plot, and the percentage of variance explained. These steps ensured a robust identification of the instrument's latent structure.

Subsequently, CFA was conducted to evaluate the model's fit to the empirical data. Normality was assessed through skewness (Sk) and kurtosis (Ku) coefficients, with values of |Sk| < 3 and |Ku| < 10 indicating no substantial deviations that would affect the use of the maximum likelihood estimation method. The Mahalanobis squared distance (D^2^) was used to detect potential outliers. Given the low incidence of missing data (<4%), mean imputation was applied for the corresponding variables.

Model fit was evaluated using several goodness-of-fit indices, with the following thresholds considered acceptable: χ^2^/df < 5, CFI and GFI > 0.90, and RMSEA <0.08. The model with the strongest external validity was selected based on the lowest MECVI value. Further refinements were guided by modification indices (MI), using a threshold of MI > 11 and a significance level of *p* < 0.001, supported by theoretical justification. The reliability of the instrument was assessed using Cronbach's alpha (α), McDonald's omega (ω), and composite reliability (CR). Values above 0.70 were considered indicative of acceptable internal consistency across indices. Construct validity was evaluated through three components: (i) convergent validity, assessed via the average variance extracted (AVE) for each factor, with values above 0.50 indicating adequacy; (ii) discriminant validity, confirmed when the AVE of any two factors was greater than or equal to the squared correlation between them; and (iii) factorial validity, examined through standardized factor loadings (λ) and individual item reliability (λ^2^), with recommended thresholds of λ > 0.50 and λ^2^ > 0.25.

This study followed the recommendations by DeVellis and Thorpe [31], Lloret-Segura et al. [32], and Ferrando et al. [33]. Descriptive analyses, including measures of central tendency, dispersion, and frequency, as well as the EFA, were performed using the Statistical Package for the Social Sciences (SPSS, version 29.0; IBM, Chicago, IL). CFA and invariance testing were conducted using AMOS software (version 22.0; IBM, Chicago, IL).

## 3 Results

### 3.1 Cross-cultural adaptation

During the expert consensus meeting for Version 1, the panel unanimously agreed to adopt the term “the chosen option” as a translation for both “the option I chose” and “the choice I made.” This translation was considered to more accurately convey the concept of selecting among multiple possibilities, thus better aligning with the original intent of the scale. Pretesting was conducted with 18 pregnant women enrolled in a Childbirth Preparation Program (CPP). The average time to complete the instrument was 12 min. During follow-up interviews, nulliparous women reported greater difficulty answering certain questions related to others' reactions and hypothetical situations they had not yet encountered. These challenges were addressed by adding more specific guidance in the questionnaire's preamble. Additionally, participants suggested replacing the term “hospital (clinic)” with “health service/institution,” which was regarded as more culturally appropriate and widely understood in the Portuguese context.

### 3.2 Sample characteristics

The study included 241 pregnant women enrolled in Childbirth Preparation Programs (CPPs) at nine Community Care Units (CCUs) in central Portugal, between September 2023 and September 2024. Participants ranged in age from 20 to 45 years, with a mean age of 32.2 years (SD = 4.8). Gestational age varied from 21 to 41 weeks, with a mean of 33.5 weeks (SD = 3.8). The majority of participants (72.6%) were nulliparous, while 21.1% had experienced at least one previous birth. Most women (65.1%) had completed higher education, with 41.9% holding a bachelor's degree. Only 4.1% had an education level below the 12th grade. Regarding employment status, most participants (91.3%) were employed, and among them, 79.7% worked as salaried employees. Additionally, the vast majority (96.3%) reported living with the baby's father in a cohabiting arrangement. There were no missing values for any of the variables analyzed (Table 1).

**Table 1:** Sociodemographic and obstetric characteristics of the sample (*n* = 241).

Variable	Mean (±SD)	Range	n (%)
**Age (years)**	32.2 (± 4.7)	20–45	
**Gestational age (weeks)**	33.5 (± 3.8)	21–41	
**Educational qualifications**			
Less than secondary education			10 (4.1)
Secondary education			74 (30.7)
Bachelor's degree			101 (41.9)
Master's degree			53 (22.0)
Doctorate			3 (1.2)
**Employment status**			
Self-employed			28 (11.6)
Employed (salaried)			192 (79.7)
Unemployed			19 (7.9)
Student			2 (0.8)
**Household composition**			
Lives alone			5 (2.1)
Lives with a partner			163 (67.6)
Lives with a partner and child(ren)			65 (27.0)
Lives with other family members			4 (1.7)
Lives with a partner and other family members			4 (1.7)
**Number of previous births**			
Zero			175 (72.6)
One			51 (21.2)
Two			12 (5.0)
Three or more			3 (1.2)

*Note.* SD = standard deviation.

### 3.3 Psychometric properties

#### 3.3.1 Exploratory factor analysis

The total sample of 241 women was randomly divided for analysis purposes, with exploratory factor analysis (EFA) conducted on a subsample of 110 participants and confirmatory factor analysis (CFA) performed on a separate subsample of 131 participants.

In the EFA subsample (*n* = 110), descriptive item analysis indicated adequate psychometric sensitivity for factor analysis. Item-level distributional indices (skewness and kurtosis) were computed and showed values within acceptable limits (|Sk| < 3; |Ku| < 10), confirming the absence of substantial deviations from normality. These findings support the use of Pearson correlations in the EFA and the application of maximum likelihood estimation in the subsequent CFA. The data's suitability was supported by the Kaiser-Meyer-Olkin (KMO) measure, which indicated good sampling adequacy (KMO = 0.80), and by Bartlett's test of sphericity, which confirmed significant inter-item correlations (*p* < 0.001). Based on the eigenvalue >1 criterion and inspection of the scree plot, five latent factors were retained, accounting for 65.71% of the total variance. Communality values were high, ranging from 0.42 to 0.80, indicating that the retained factors effectively represented the underlying correlational structure.

Most items demonstrated strong factor loadings (> 0.50) on a single factor, supporting the presence of a coherent factorial structure. However, Item 1 exhibited its highest loading on factor 3 (0.39) but also displayed similar cross-loadings on other factors. This pattern suggests that Item 1 may lack sufficient discriminant validity and does not clearly align with any specific latent dimension (Table 2). Given these findings, CFA was deemed essential to evaluate the adequacy of the proposed factor structure.

**Table 2:** Factor loading from the exploratory factorial analysis.

					Components				
	N	Mean ± SD	Sk	Ku	1	2	3	4	5
1. A opção que escolhi é a que penso que a maioria das mulheres escolhe *The option I chose is the one I think most women choose*	108	3.22 ± 0.84	−0.33	0.21			0.39		
2. A minha escolha foi influenciada pela pressão de outras pessoas na minha vida *My choice was made based on pressure from others in my life*	108	4.25 ± 0,79	−1.02	1.35					0.77
3. A opção que escolhi foi a melhor opção para mim *The option I chose was the best option for me*	108	4.07 ± 0.72	−0.13	−1.05	0.59				
4. Senti-me pressionada pelos profissionais de saúde para fazer a escolha que fiz *I felt pressure from health professionals to choose the option I chose*	108	4.07 ± 0.83	−0.36	−0.95					0.68
5. A opção que escolhi adequa-se ao meu estilo de vida *The option I chose fits my lifestyle*	108	3.82 ± 0.78	−0.74	1.73	0.76				
6. A opção que escolhi adequa-se a mim *The option I chose suits me personally*	108	3.97 ± 0.74	−0.79	1.78	0.77				
7. A opção que escolhi é a melhor *The option I chose is the best*	108	3.55 ± 0.73	0.37	−0.32	0.72				
8. A opção que escolhi por vezes provoca reações negativas nos enfermeiros e/ou médicos *The option I chose sometimes produces negative reactions from the nurses and/or doctors*	108	3.33 ± 0.82	0.24	0.15				0.65	
9. Pressionei-me a mim mesma para fazer esta escolha *I put pressure on myself to choose this option*	108	4.06 ± 0.85	−0.89	0.96					0.50
10. Escolhi esta opção sem que ninguém me pressionasse *I chose this option without pressure from anyone else*	108	3.99 ± 0.92	−1.26	1.69					0.68
11. A escolha que fiz é a melhor para mim *The choice I made is the best one for me*	108	3.91 ± 0.76	−0.12	−0.65	0.73				
12. Todos estiveram disponíveis para me ajudar a fazer a minha escolha *Everyone has been supportive in helping me make my choice*	108	3.72 ± 0.89	−0.66	0.56			0.80		
13. Penso que o serviço/instituição de saúde me ajudou a manter a minha escolha *I think the hospital (clinic) helped me to maintain my choice*	108	3.62 ± 0.87	−0.36	0.32			0.74		
14. Havia pessoas à minha disposição no serviço/instituição de saúde para me ajudar a decidir *There were people available to me in the hospital (clinic) to help me decide*	108	3.77 ± 0.92	−0.62	0.33			0.85		
15. A opção que escolhi por vezes provoca reações negativas nos meus amigos *The option I chose sometimes produces negative reactions from my friends*	108	3.61 ± 0.94	−0.06	−0.89				0.81	
16. A opção que escolhi por vezes provoca reações negativas na minha família *The option I chose sometimes produces negative reactions from my family*	108	3.76 ± 0.90	−0.20	−0.80				0.84	
17. Sinto-me bem com esta escolha *I feel good about this option*	108	4.18 ± 0.73	−1.02	2.47		0.78			
18. Vou manter a escolha que fiz *I will stick to the option I chose*	108	4.03 ± 0.84	−0.74	0.69		0.75			
19. A minha família apoiou a minha decisão *My family supported my decision*	108	3.92 ± 0.83	−0.15	−0.94		0.64			
20. Foi fácil para mim decidir *It was easy for me to decide*	108	3.86 ± 0.83	−0.91	1.59		0.57			
Variance (%)					16.3%	13.7%	12.3%	12.0%	11.5%

*Note.* N = sample size; SD = standard deviation; Sk = Skewness; Ku = kurtosis.

Exploratory factor analysis using Principal Axis Factoring with oblique Promax rotation identified five factors with eigenvalues greater than 1, collectively accounting for 65.8% of the total variance. The resulting factor solution was interpretable and theoretically coherent, with items showing salient loadings on their respective factors, supporting a well-defined factorial structure.

#### 3.3.2 Confirmatory factor analysis

**Fig. 1:**
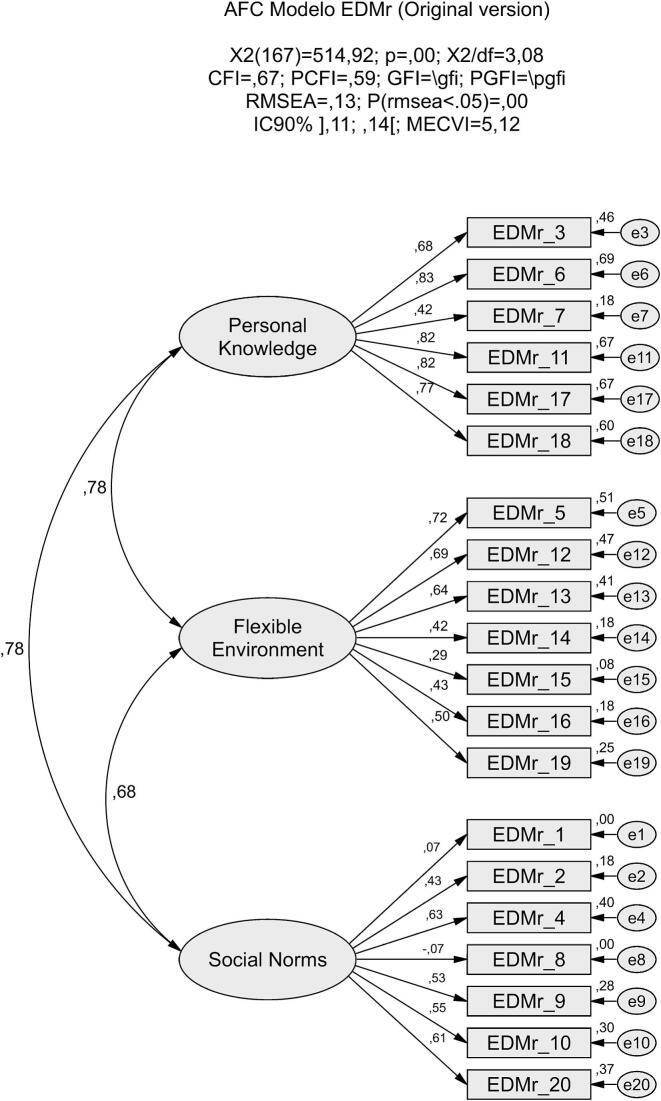
Confirmatory factor model of the Portuguese version of the EDMr, showing the original three-factor structure.

**Fig. 2:**
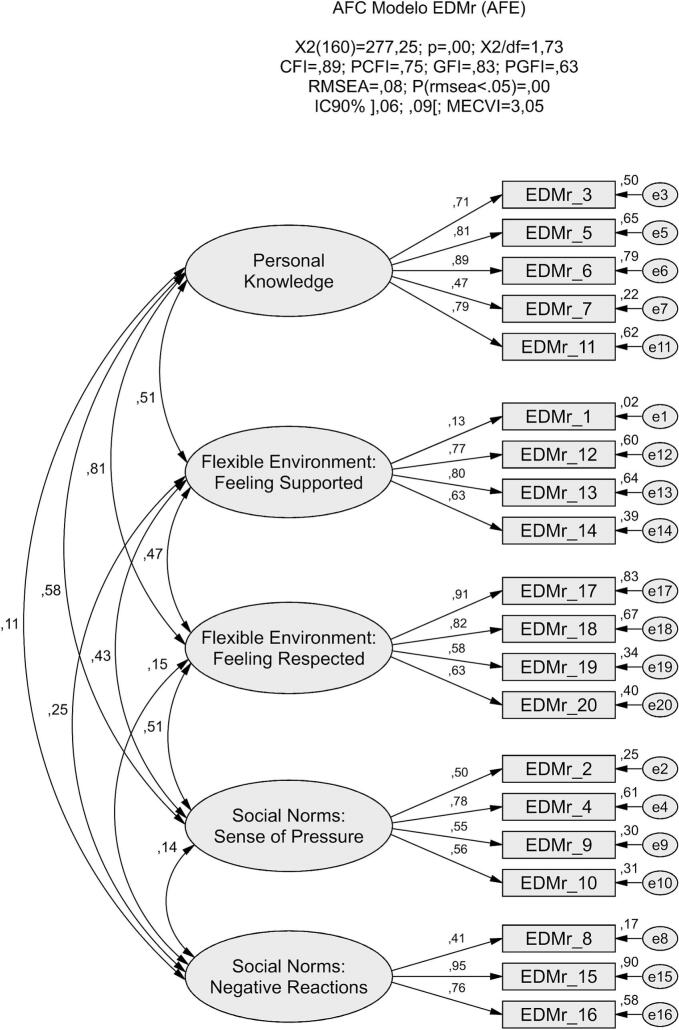
Confirmatory representation of the five-factor model of the Portuguese version of the EDMr, derived from the exploratory factor analysis.

Confirmatory factor analysis (CFA) was performed on a subsample of 131 women to evaluate model fit, considering both the original theoretical model (Fig. 1) and the model derived from the exploratory factor analysis (EFA) results (Fig. 2). The goodness-of-fit indices demonstrated that the EFA-derived model provided a significantly superior fit to the data compared to the original model.

The analysis indicated an overall acceptable model fit across most indices (χ^2^/df = 1.73; CFI = 0.89; GFI = 0.83; RMSEA = 0.08; MECVI = 3.05). Standardized factor loadings (λ) and individual item reliabilities (λ^2^) were generally adequate. However, Item 1 (“The option I chose is the one I think most women choose”) exhibited a very low factor loading (λ = 0.13), consistent with findings from the EFA, which led to its exclusion from the model. Items 7 (λ = 0.47) and 8 (λ = 0.41) also showed loadings below the recommended threshold. Nonetheless, following a conservative approach and considering their theoretical relevance, these items were retained in the model.

**Fig. 3:**
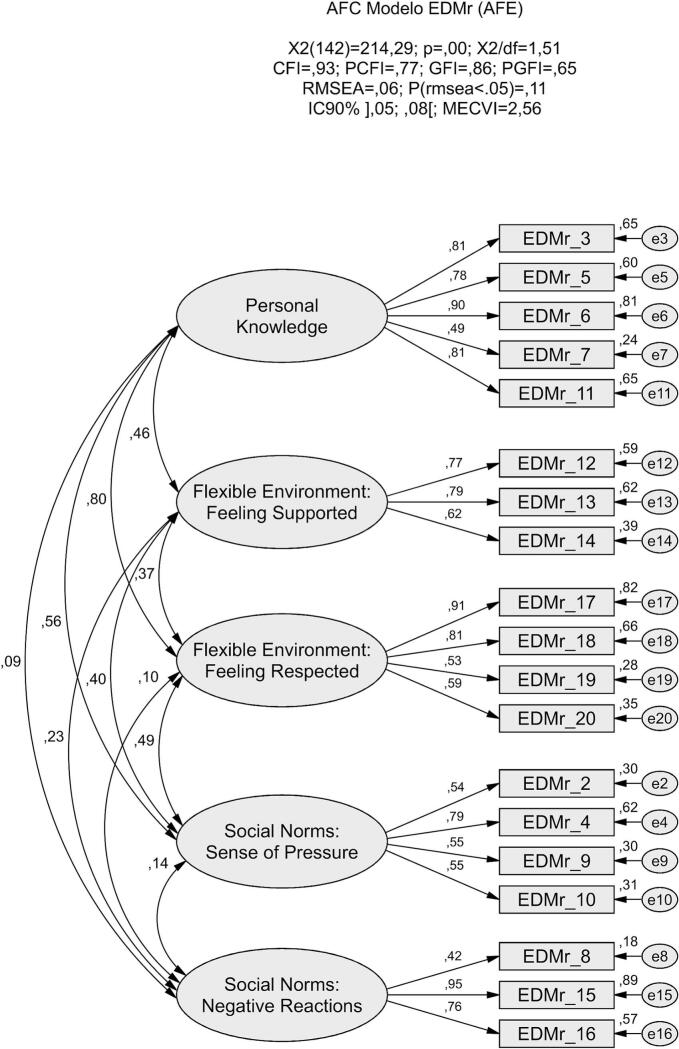
Refined five-factor model of the Portuguese version of the EDMr, confirmed through confirmatory factor analysis (CFA) with standardized loadings and factor correlations.

All items met the assumptions for univariate normality. However, multivariate outliers were detected (p₁ and p₂ < 0.001), prompting a reanalysis after excluding two cases with elevated Mahalanobis distances (D^2^). This adjustment resulted in improved model fit indices (χ^2^/df = 1.51; CFI = 0.93; GFI = 0.86; RMSEA = 0.06; MECVI = 2.56), as presented in Fig. 3.

The model fit of the final five-factor structure was significantly superior to that of the initial solution (χ^2^ (18) = 62.96, *p* < 0.05), as evidenced by a lower MECVI value (MECVI = 2.56), indicating better external validity. As shown in Table 3, construct reliability was adequate for most dimensions, with both composite reliability (CR) and Cronbach's alpha (α) values equal to or exceeding 0.70. The exception was the *Social Norms: Sense of Pressure* factor, which presented a slightly lower alpha coefficient (α = 0.69). Standardized factor loadings ranged from 0.42 to 0.94, while individual item reliability (λ^2^) ranged from 0.18 to 0.90. Convergent validity was acceptable for all dimensions except *Social Norms: Sense of Pressure*, which showed a lower average variance extracted (AVE = 0.38). Discriminant validity was generally supported through comparison between AVE values and the squared correlations among factors. However, overlap was observed between the *Personal Knowledge* and *Flexible Environment: Feeling Respected* dimensions, suggesting limited discriminant validity between these two constructs.

**Table 3:** Construct reliability, convergent validity, and discriminant validity for the EDMr-PT.

Factors	Number of items	CR	α	AVE	ρ^2^
Personal Knowledge	5	0.88	0.86	0.59	0.01–0.64
Flexible Environment: Feeling Supported	3	0.77	0.77	0.53	0.05–0.21
Flexible Environment: Feeling Respected	4	0.81	0.78	0.53	0.01–0.64
Social Norms: Sense of Pressure	4	0.70	0.69	0.38	0.02–0.31
Social Norms: Negative Reactions	3	0.77	0.72	0.55	0.01–0.05

*Note.* CR = Composite reliability; α = Cronbach's alpha; AVE = average variance extracted; ρ^2^ = squared correlation between factors; EDMr-PT = Portuguese Version of the Revised Emancipated Decision-Making Scale.

To further validate the factorial structure of the EDMr-pt, an additional exploratory factor analysis (EFA) was conducted using the full sample (*N* = 241) to examine the stability and replicability of the structure previously identified in the separate EFA and CFA subsamples. The results revealed excellent sampling adequacy (KMO = 0.859; *p* < 0.001) and supported the extraction of five factors with eigenvalues greater than 1, jointly explaining 64.07% of the total variance. Exploratory factor analysis was performed using a common-factor extraction method (Principal Axis Factoring) with oblique Promax rotation, which is theoretically appropriate given the expected correlations among latent dimensions. The pattern matrix showed a coherent distribution of items across the five factors, with loadings above 0.40 and conceptually consistent groupings. This structure fully replicated the previously identified five-factor solution, thereby reinforcing the factorial validity of the Portuguese version of the scale.

Although Item 1 (*The option I chose is the one I think most women choose*) presented an acceptable factor loading (0.615) in this full-sample EFA, it was not reintegrated into the final version of the scale. This decision was based on its lack of stability in previous analyses: in the initial EFA (*N* = 110), it did not present a significant loading, and in the subsequent CFA (*N* = 131), it showed a very low standardized loading. For reasons of methodological consistency and empirical robustness, only the 19 items that consistently demonstrated both statistical and conceptual alignment across all validation stages were retained.

The decision to adopt the structure derived from the full-sample EFA (*N* = 241) as the final validated model is justified by its greater statistical stability, excellent internal consistency, and complete replication of the factor structure previously identified. This analysis does not replace the earlier results but rather reinforces them, providing stronger evidence for the reliability and validity of the Portuguese version of the EDMr-r and supporting its future application in both clinical and research settings.

Internal consistency was evaluated at the subscale level, as recommended for multidimensional instruments. Cronbach's alpha (α) and McDonald's omega (ω) coefficients indicated acceptable to high reliability across dimensions: Personal Knowledge (α = 0.86, ω = 0.83), Feeling Supported (α = 0.79, ω = 0.75), Feeling Respected (α = 0.81, ω = 0.79), Sense of Pressure (α = 0.68, ω = 0.79), and Negative Reactions (α = 0.72, ω = 0.73). For comparison, the original three-factor model reported alpha coefficients of α = 0.88 (Personal Knowledge), α = 0.72 (Flexible Environment), and α = 0.57 (Social Norms), along with McDonald's ω values of 0.87, 0.74, and 0.58, respectively. These comparisons indicate that the Portuguese adaptation preserves the theoretical core of the EDMr while showing improved internal consistency in several dimensions.

Together, these findings support the reliability, conceptual coherence, and statistical stability of the final five-factor structure of the EDMr-pt.

## 4 Discussion and conclusion

### 4.1 Discussion

This study aimed to adapt and validate the EDMr scale for the Portuguese context, contributing to the global effort to assess women's autonomy in healthcare decision-making, particularly in maternity care. The cross-cultural adaptation followed the guidelines proposed by Beaton et al. [29], and the psychometric evaluation involved both Exploratory Factor Analysis (EFA) and Confirmatory Factor Analysis (CFA).

The initial analysis considered the original three-factor structure of the scale. However, the results supported the subdivision of two components, resulting in a refined five-factor model. This restructuring improves the scale's conceptual sensitivity and contextual alignment, ensuring greater cultural relevance and psychometric robustness within the Portuguese population.

The Personal Knowledge component now consists of five items (3, 5, 6, 7, and 11). Four of these were originally part of the same subscale, while item 5 was reassigned from the Flexible Environment component. This reassignment is theoretically coherent, as it emphasizes the woman's evaluation of how the decision aligns with her personal circumstances, lifestyle, and values [5,25].

The original Flexible Environment subscale, composed of seven items, was divided into two more specific components. The first, *Flexible Environment: Feeling Supported*, includes items 12, 13, and 14. The second, *Flexible Environment: Feeling Respected*, includes items 17, 18, 19, and 20. Items 17 and 18 were reassigned from the Personal Knowledge component, and item 20 from the Social Norms component. This theoretical reorganization highlights two key aspects of a flexible environment: on the one hand, the support and availability perceived by women in making their decisions, and on the other, the respect, validation, and emotional security they feel regarding those decisions. It reflects their sense of comfort, confidence, and autonomy throughout the decision-making process [5,25].

The Social Norms component was also subdivided into two dimensions. *Social Norms: Sense of Pressure* includes items 2, 4, 9, and 10. *Social Norms: Negative Reactions* includes items 8, 15, and 16, originally from the Flexible Environment component. This new structure reinforces two dimensions of perceived social influence: first, how societal and interpersonal pressures shape women's choices; second, how their decisions may trigger negative judgments or disapproval from others. Together, these dimensions provide a more nuanced understanding of how social norms affect women's autonomy in healthcare settings.

Numerous studies conducted in developing countries have highlighted the persistent gap between the theoretical recognition of women's right to self-determination and its practical implementation. Although women's autonomy is widely affirmed in policy and public discourse, conservative and paternalistic norms often prevail, limiting their actual ability to make emancipated healthcare decisions [11,17]. However, these challenges are not unique to low-resource settings. In high-income countries, similar patterns persist, such as restricted access to information, limited choices, and the continued dominance of healthcare professionals in decision-making, which collectively contribute to inadequately informed consent [[18], [19], [20]]. For example, a study conducted in Ireland revealed how contextual and relational dynamics, such as pressure to accept medical interventions, disagreements with healthcare providers, and insufficient professional support, can significantly undermine women's participation in decision-making during labor [21].

A recent systematic review identified multiple factors influencing women's autonomy in healthcare decision-making, including age, level of education, occupation, partner's education and occupation, region of residence, household wealth, cultural background, and religious beliefs. Improving women's decision-making autonomy requires coordinated efforts across sectors. Policymakers play a pivotal role by enacting legislation that protects women's rights, promoting gender-sensitive healthcare services, ensuring equitable access to comprehensive health information, expanding health education initiatives, and developing targeted support for vulnerable populations [34].

In this study, Item 1, originally part of the Social Norms subscale, was excluded due to factor dispersion and low factor loading in the confirmatory factor analysis (CFA). This exclusion raises important considerations. One possible explanation is that, in the context of childbirth, women may not compare their decisions to those of others, given the deeply personal, emotionally intense, and time-sensitive nature of the experience. Additionally, many participants were nulliparous and therefore lacked prior birth experiences for comparison. As a result, they may not perceive options such as mobility or upright positioning during labor as autonomous choices, but rather as clinical decisions determined by healthcare professionals. Even when women are aware of available options, doubts often remain about whether their preferences will be respected upon hospital admission. This uncertainty may contribute to feelings of disempowerment and diminished autonomy in the childbirth experience.

The clinical scenario selected for this study may have further influenced these perceptions. For nulliparous women, in particular, the concept of mobility during labor may be unfamiliar or perceived as irrelevant, due to limited experience or awareness. Additionally, the use of both present and past tense in item wording may have contributed to confusion, as well as the inclusion of reverse-scored items (2, 4, 8, 9, 15, 16), which are known to potentially reduce clarity and psychometric performance [35].

Finally, broader sociocultural and political differences between the original (U.S.) and target (Portuguese) contexts should be considered. These likely influenced the substantial reorganization of items and the emergence of a new five-factor model in the Portuguese version of the EDMr.

Notably, this is the first validation study of the EDMr scale conducted outside the United States. As such, no cross-cultural psychometric comparisons are currently available. Nonetheless, internal consistency findings can be compared with those reported in the original study. Wittmann-Price and Price [28] reported a Cronbach's alpha of 0.89 for the total scale, with subscale values of 0.74 for Flexible Environment, 0.78 for Personal Knowledge, and 0.77 for Social Norms. These figures are largely comparable to those obtained in the present study, where all subscales met or exceeded the commonly accepted threshold of 0.70 for adequate internal consistency [31], with the exception of one Social Norms subscale (α = 0.69).

The Portuguese version of the EDMr demonstrated strong internal consistency, with a Cronbach's alpha of 0.87 for the total scale. Among the subscales, the Personal Knowledge subscale showed higher reliability (α = 0.86) compared to the original version (α = 0.78). Similarly, both dimensions of the Flexible Environment subscale showed slightly improved internal consistency (α = 0.77 and 0.78, respectively, compared to 0.74 in the original version). In contrast, the Social Norms subscale exhibited lower reliability in the Portuguese version (α = 0.72 and 0.69) relative to the original (α = 0.77). These findings support the overall reliability of the EDMr scale across cultural contexts while also suggesting that cultural nuances may influence how social influences are perceived and measured. This interpretation is consistent with evidence from other instruments assessing patient empowerment and autonomy, such as the Shared Decision-Making Questionnaire (SDM-Q-9) [36] and the Patient Activation Measure (PAM) [37]. Both instruments, originally developed in the United States and later adapted to multiple cultural contexts, emphasize that decision-making autonomy is shaped not only by individual knowledge and confidence but also by relational and contextual dynamics within healthcare encounters. Cross-cultural validations of the SDM-Q-9 have shown that cultural values and healthcare hierarchies influence how patients perceive their right and ability to participate in decisions, while the Portuguese adaptation of the PAM-13 [38] demonstrated that patient activation is affected by sociocultural expectations, health literacy, and healthcare system characteristics. These parallels reinforce that emancipated decision-making, like empowerment and shared decision-making, cannot be fully understood without acknowledging its cultural embeddedness.

The relatively low internal consistency (α = 0.69) and average variance extracted (AVE = 0.38) for the Sense of Pressure subscale suggest that item refinement or the addition of new items could be explored in future research to improve construct stability and validity.

Several limitations should be acknowledged. First, despite a rigorous cross-cultural adaptation process, certain cultural subtleties, especially among women at different life stages or with varying healthcare experiences, may not have been fully captured, affecting the sensitivity of some items. Second, the study relied on a convenience sample of women recruited from nine community care units in primary healthcare settings in central Portugal, which may have introduced selection bias and limits the generalizability of the findings to other regions or levels of care. Third, although the total sample size (*N* = 241) met classical recommendations for factor analysis, dividing it into two subsamples for Exploratory and Confirmatory Factor Analyses may have affected the stability of the factor solutions. Future research with larger and more diverse samples is recommended to confirm the robustness of the identified structure. Regarding unidimensionality, this was not tested in the present study because the EDMr is theoretically grounded in a multidimensional framework comprising interrelated but distinct domains of women's decision-making. However, future studies may test a single-factor model to further examine construct distinctiveness. Future research with larger and more diverse samples is recommended to confirm the robustness of the identified structure. Additionally, the clinical scenario used to frame responses may have introduced interpretive challenges, particularly for nulliparous women responding to items related to labor and delivery decisions. Expanding the scale's application to other healthcare decision-making contexts would also help to establish its broader cross-cultural utility.

### 4.2 Innovation

This study offers a novel psychometric contribution by adapting and validating the EDMr scale for use in the Portuguese context, representing the first such initiative conducted outside the United States. Beyond a straightforward linguistic translation, the study involved a critical reexamination of the scale's factorial structure, leading to the development of a refined five-factor model. This methodological innovation improves the instrument's alignment with Portuguese sociocultural norms and enhances its conceptual clarity in a new healthcare environment.

More generally, the research extends the application of Emancipated Decision-Making Theory to a distinct cultural and healthcare system, providing new theoretical and empirical insights into how women's autonomy is shaped in Southern European maternity care. The study exemplifies the innovative cross-cultural adaptation of psychometric tools and supports their use in international and comparative health research. This dual framing, conceptual expansion and methodological refinement, lays a solid foundation for future applications of the EDMr in other cultural contexts and healthcare systems.

## 5 Conclusion

This study indicates that the refined five-factor structure of the Portuguese version of the EDMr (EDMr-PT) demonstrates strong theoretical alignment and cultural relevance. Its use in the childbirth context provides valuable insights into how women perceive autonomy, professional support, and social influence in healthcare decision-making. These findings highlight the importance of promoting respectful, woman-centered maternity care and underscore the critical role of nursing and midwifery interventions in facilitating emancipated decisions. Accordingly, the EDMr-PT emerges as a practical and meaningful tool for advancing both research and clinical practice in maternal healthcare and women's rights. Moreover, the scale holds significant potential for future cross-cultural comparative studies, contributing to a deeper, more global understanding of women's decision-making experiences across varied healthcare systems.

## CRediT authorship contribution statement

**Marlene Isabel Lopes:** Validation, Software, Project administration, Methodology, Investigation, Formal analysis, Data curation, Conceptualization, Writing – review & editing, Writing – original draft. **Teresa Margarida Almeida Neves:** Validation, Software, Formal analysis, Writing – original draft. **Ruth A. Wittmann-Price:** Validation, Supervision, Conceptualization, Writing – review & editing. **Margarida Vieira:** Supervision, Conceptualization, Writing – review & editing. **Alexandrina Cardoso:** Validation, Supervision, Formal analysis, Conceptualization, Writing – review & editing.

## Funding statement

Not applicable.

## Declaration of competing interest

The authors declare that they have no known competing financial interests or personal relationships that could have appeared to influence the work reported in this paper.

## References

[1] Elwyn G., Frosch D., Thomson R. (2012). Shared decision making: a model for clinical practice. J Gen Intern Med.

[2] Bawafaa E. (2024). Marginalization and women’s healthcare in Ghana: incorporating colonial origins, unveiling women’s knowledge, and empowering voices. Nurs Inq.

[3] Granek L., Nakash O. (2017). The impact of militarism, patriarchy, and culture on Israeli women’s reproductive health and well-being. Int J Behav Med.

[4] Obeidat R.F. (2015). Promoting emancipated decision-making for surgical treatment of early stage breast cancer among Jordanian women. Asia Pac J Oncol Nurs.

[5] Wittmann-Price R.A. (2004). Emancipation in decision-making in women’s health care. J Adv Nurs.

[6] Wittmann-Price R.A., Bhattacharya A. (2008). Reexploring the subconcepts of the Wittmann-Price theory of emancipated decision making in women’s healthcare. ANS Adv Nurs Sci.

[7] O’Connor A.M., Légaré F., Stacey D. (2003). Risk communication in practice: the contribution of decision aids. BMJ.

[8] American College of Nurse-Midwives (2016). Shared decision making in midwifery care. http://midwife.org/ACNM/files/ACNMLibraryData/UPLOADFILENAME/000000000305/Shared-Decision-Making-inMidwifery-Care-10-13-17.pdf.

[9] Alruwaili T.A., Crawford K., Jahanfar S., Hampton K., Fooladi E. (2023). Pregnant persons and birth partners’ experiences of shared decision-making during pregnancy and childbirth: an umbrella review. Patient Educ Couns.

[10] (2018). WHO recommendations: Intrapartum care for a positive childbirth experience.

[11] Negash W.D., Kefale G.T., Belachew T.B., Asmamaw D.B. (2023). Married women decision making autonomy on health care utilization in high fertility sub-Saharan African countries: a multilevel analysis of recent Demographic and Health Survey. PLoS One.

[12] Pennington A., Orton L., Nayak S. (2018). The health impacts of women’s low control in their living environment: a theory-based systematic review of observational studies in societies with profound gender discrimination. Health Place.

[13] Richards N.K., Crockett E., Morley C.P., Levandowski B.A. (2020). Young women’s reproductive health conversations: roles of maternal figures and clinical practices. PLoS One.

[14] Shai A., Koffler S., Hashiloni-Dolev Y. (2021). Feminism, gender medicine and beyond: a feminist analysis of “gender medicine”. Int J Equity Health.

[15] UNFPA (2020). https://www.unfpa.org/sites/default/files/pub-pdf/UNFPA-SDG56%201562Combined-v4.%2015.Pdf.

[16] Salinger A.P., Vermes E., Waid J.L. (2024). The role of self-efficacy in women’s autonomy for health and nutrition decision-making in rural Bangladesh. BMC Public Health.

[17] Olwanda E., Opondo K., Oluoch D. (2024). Women’s autonomy and maternal health decision making in Kenya: implications for service delivery reform - a qualitative study. BMC Womens Health.

[18] Healy S., Humphreys E., Kennedy C. (2017). A qualitative exploration of how midwives’ and obstetricians’ perception of risk affects care practices for low-risk women and normal birth. Women Birth.

[19] Hunter A., Devane D., Houghton C., Grealish A., Tully A., Smith V. (2017). Woman-centred care during pregnancy and birth in Ireland: thematic analysis of women’s and clinicians’ experiences. BMC Preg Childbirth.

[20] Huschke S. (2022). The system is not set up for the benefit of women’: women’s experiences of decision-making during pregnancy and birth in Ireland. Qual Health Res.

[21] O’Brien D., Butler M.M., Casey M. (2021). The importance of nurturing trusting relationships to embed shared decision-making during pregnancy and childbirth. Midwifery.

[22] SSD Silva, Fortuna C.M., Monceau G. (2021). Cesarean childbirth: an institutional socio-clinical study of the professional practices and discourses. Rev Lat Am Enfermagem.

[23] Breman R.B., Waddell A., Watkins V. (2024). Shared decision making in perinatal care. J Obstet Gynecol Neonatal Nurs.

[24] Lopes M.I., Wittmann-Price R.A. (2025). The Wittmann-Price theory of emancipated decision-making in women’s health care: an analysis based on McEwen. Holist Nurs Pract.

[25] Wittmann-Price R.A. (2006). Exploring the subconcepts of the Wittmann-Price theory of emancipated decision-making in women’s health care. J Nurs Scholarsh.

[26] Wittmann-Price R.A., Fliszar R., Bhattacharya A. (2011). Elective cesarean births: are women making emancipated decisions?. Appl Nurs Res.

[27] Stepanuk K.M., Fisher K.M., Wittmann-Price R., Posmontier B., Bhattacharya A. (2013). Women’s decision-making regarding medication use in pregnancy for anxiety and/or depression. J Adv Nurs.

[28] Wittmann-Price R.A., Price S.W. (2014). Development and revision of the Wittmann-Price emancipated decision-making scale. J Nurs Meas.

[29] Beaton D., Bombardier C., Guillemin F., Ferraz M.B. (2007).

[30] Boateng G.O., Neilands T.B., Frongillo E.A., Melgar-Quiñonez H.R., Young S.L. (2018). Best practices for developing and validating scales for health, social, and behavioral research: a primer. Front Public Health.

[31] DeVellis R., Thorpe C. (2017).

[32] Lloret-Segura S., Ferreres-Traver A., Hernández-Baeza A., Tomás-Marco I. (2014). Exploratory item factor analysis: a practical guide revised and updated. Anal Psicol.

[33] Ferrando P.J., Lorenzo-Seva U., Hernández-Dorado A., Muñiz J. (2022). Decalogue for the factor analysis of test items. Psicothema.

[34] Idris I.B., Hamis A.A., ABM Bukhori (2023). Women’s autonomy in healthcare decision making: a systematic review. BMC Womens Health.

[35] Kam C.C.S. (2023). Why do regular and reversed items load on separate factors? Response difficulty vs. Item Extremity Educ Psychol Meas.

[36] Kriston L., Scholl I., Hölzel L., Simon D., Loh A., Härter M. (2010). The 9-item shared decision making questionnaire (SDM-Q-9): development and psychometric properties in a primary care sample. Patient Educ Couns.

[37] Hibbard J.H., Stockard J., Mahoney E.R., Tusler M. (2004). Development of the patient activation measure (PAM): conceptualizing and measuring activation in patients and consumers. Health Serv Res.

[38] Almeida V., Alves M., Duarte J., Ferreira P. (2019). Adaptação e validação da versão portuguesa da Patient Activation Measure (PAM-13) em pessoas com diabetes mellitus tipo 2. Acta Medica Port.

